# Giant Prostatic Stones and a Massive Bladder Stone Leading to Obstructive Uropathy: A Rare Case Report

**DOI:** 10.7759/cureus.43176

**Published:** 2023-08-08

**Authors:** Mohammad Hifzi Mohd Hashim, Suzliza Shukor, Muhammad Hasif Azizi

**Affiliations:** 1 Urology Unit, Surgical Department, Universiti Kebangsaan Malaysia Medical Centre, Kuala Lumpur, MYS

**Keywords:** spina bifida, open cystolithotomy, obstructive uropathy, bladder stone, giant prostatic stone

## Abstract

Giant prostatic calculi are rare with less than 20 cases reported in the literature so far. Here, we discuss the presentation, diagnosis, and surgical management of a 25-year-old male patient with giant prostatic stones associated with a large bladder stone resulting from an underlying neurogenic bladder secondary to spina bifida. The patient had a history of congenital spina bifida, hydrocephalus, and non-compliance with clean intermittent self-catheterization. The stones were diagnosed through imaging and cystoscopy, and open cystolithotomy was performed for stone removal. The patient had a successful postoperative recovery with improved renal function. The case highlights the association between prostatic calculi and bladder outlet obstruction, emphasizes the importance of addressing underlying conditions to prevent stone recurrence, and underscores the role of open surgery in managing large bladder stones accompanied by renal impairment.

## Introduction

The occurrence of giant prostatic urethral calculi is rare, with fewer than 20 cases reported in the literature [1]. Furthermore, the simultaneous presence of giant prostatic and bladder calculi is extremely uncommon. Here, we present a case of a patient with a giant prostatic and bladder calculus, resulting in bilateral obstructive uropathy. This condition was successfully resolved through complete removal of both stones via open surgery.

## Case presentation

The patient, a 25-year-old male, presented with underlying congenital spina bifida (lumbar myelomeningocele) and a history of hydrocephalus, for which a ventriculoperitoneal (VP) shunt was placed at seven months of age. He also had a neurogenic bladder and was on regular clean intermittent self-catheterization (CISC). However, he defaulted from follow-up since the age of 15 and did not comply with the CISC regimen.

The patient presented to the casualty department with complaints of lethargy, poor oral intake, and reduced urine output persisting for one week. Upon examination, he appeared dehydrated and lethargic. Abdominal examinations did not reveal any significant findings, but a stony hard prostate was palpable on digital rectal examination (DRE). Blood investigations revealed acute kidney injury (urea: 25 mmol/l, creatinine: 737.9 umol/l) with severe metabolic acidosis (pH: 7.18, bicarbonate: 5.2 mol/l, base excess: -20.8 mol/l). A urinary catheter was inserted, but the urine output remained minimal.

**Figure 1:**
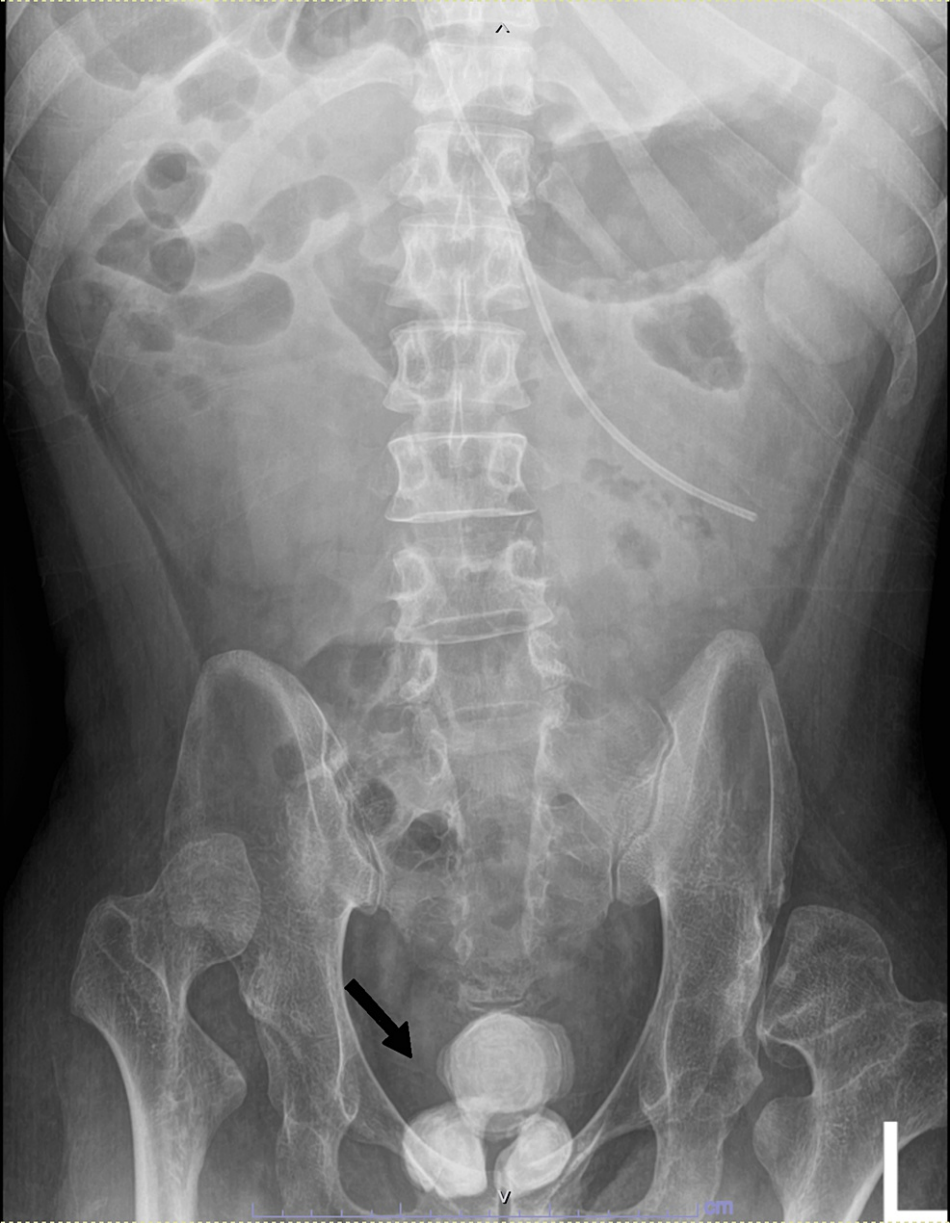
Plain X-ray kidneys, ureter, and bladder (KUB) of the patient showing two radiopaque giant prostatic stones with a single large radiopaque bladder stone (arrow).

**Figure 2:**
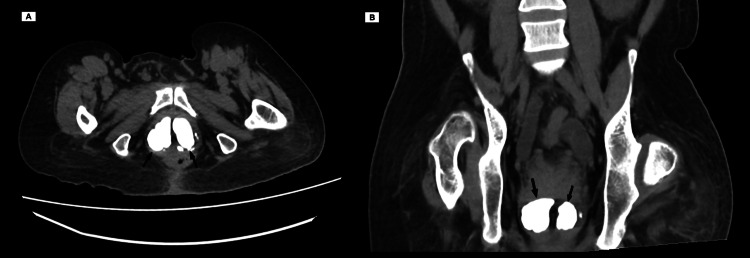
CT image in (A) axial view and (B) coronal view showing the giant prostatic stones occupying the whole prostatic gland.

**Figure 3:**
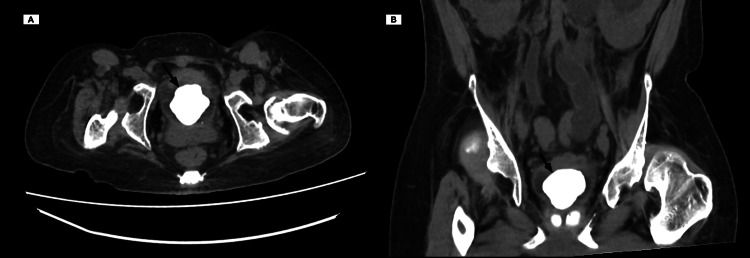
CT image in (A) axial view and (B) coronal view showing single large bladder stone occupying the whole bladder cavity.

**Figure 4:**
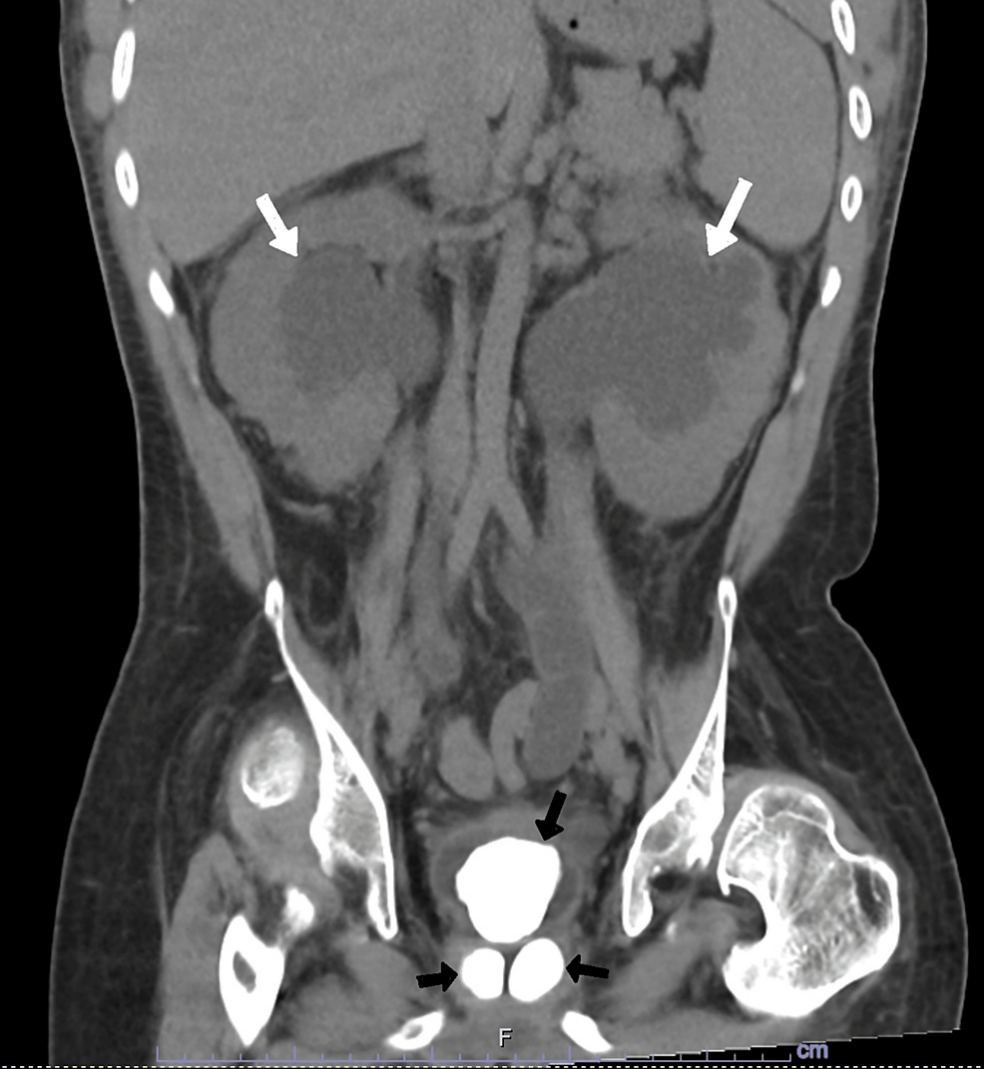
Non-contrasted CT scan image in coronal cut showing the prostatic stones occupying the gland and a large bladder stone filling the bladder (black arrows) causing bilateral gross hydronephrosis (white arrows).

**Figure 5:**
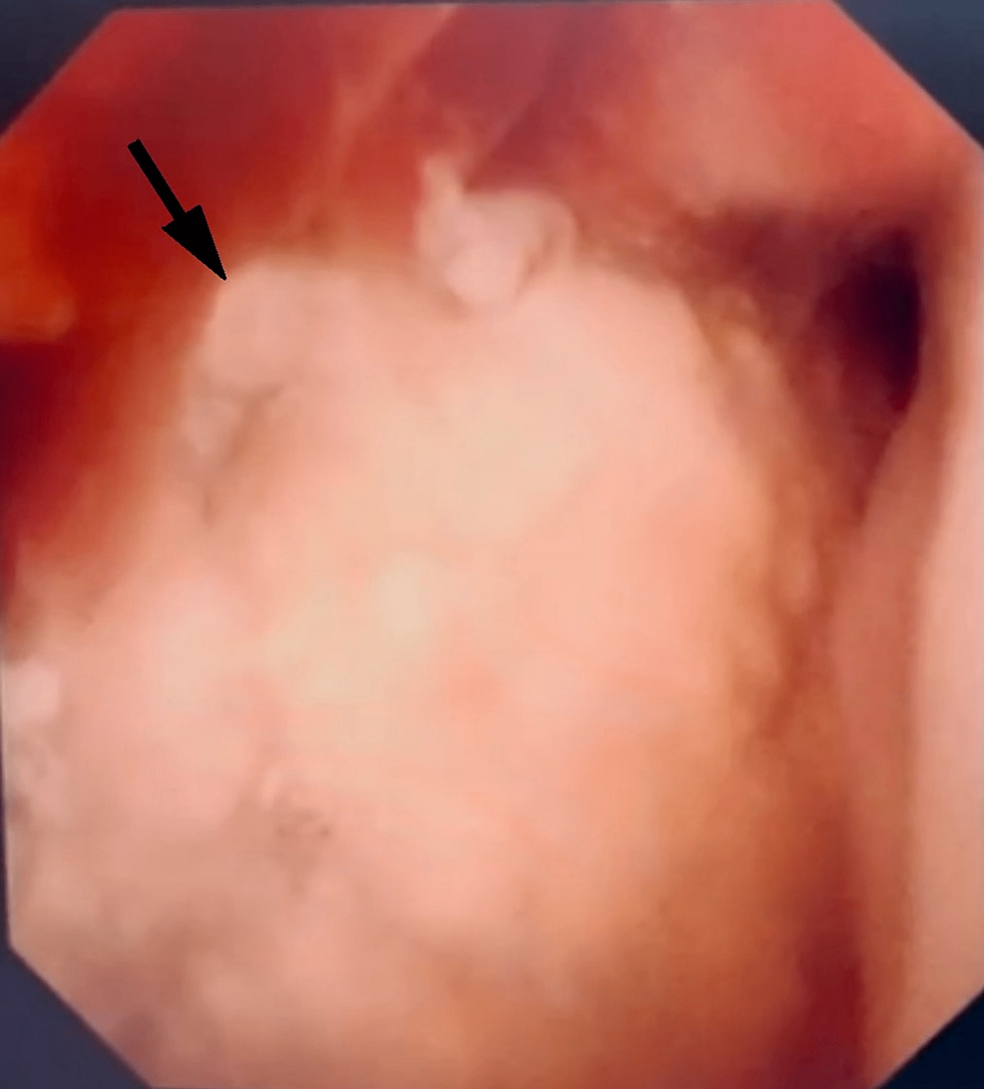
Cystoscopic view of the large bladder stone occupying the bladder cavity.

Subsequent X-ray imaging of the kidneys, ureter, and bladder (KUB) (Figure 1) and a CT scan (Figure 2-4) were performed, which revealed two prostatic stones (Figure 2) completely replacing the gland and a large bladder stone (Figure 3) causing bilateral gross hydronephrosis (Figure 4). Urethrocystoscopy examination showed no evidence of urethral stricture but revealed a large bladder stone (Figure 5) with poor visualization of both prostatic lobes. Antibiotics were commenced, and the patient was subjected for immediate surgical removal of the stones.

The patient underwent open cystolithotomy with prostatic stone extraction under general anesthesia while positioned supine. After thorough cleaning and draping, a lower midline incision was made approximately two fingers above the symphysis pubis. The anterior abdominal wall was opened in layers, and two landmarks were placed with stay sutures on the bladder. A vertical incision was then made through the detrusor muscle to access the bladder cavity. The bladder stone was completely removed in one piece using the Randall stone forceps. The prostatic stones were enucleated from the capsule through finger dissection and also removed using the same forceps and incision.

**Figure 6:**
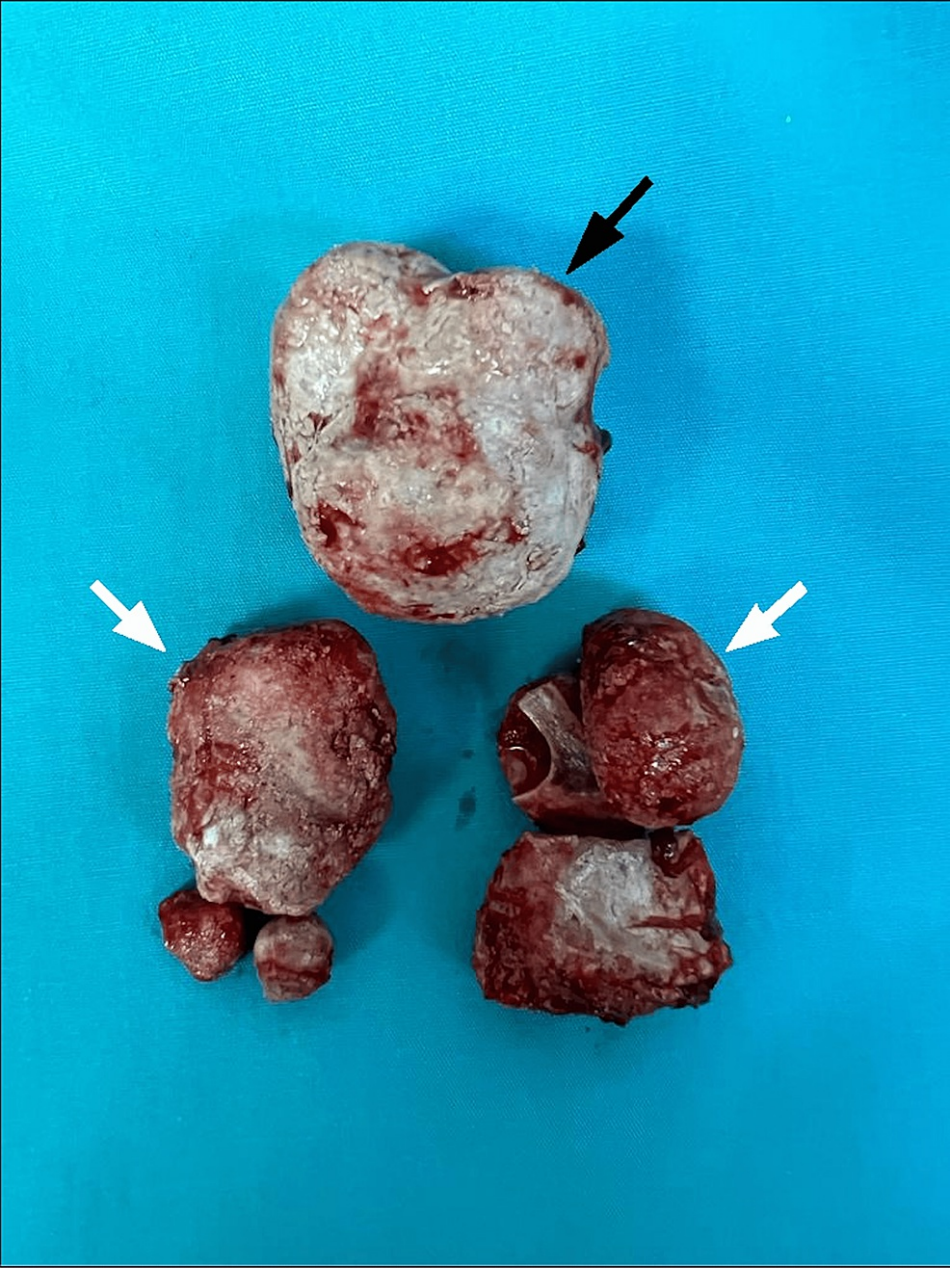
Picture of the extracted bladder stone (black arrow) and prostatic stones (white arrows).

A new suprapubic cystostomy was created, and a catheter was inserted before performing a watertight closure of the bladder using polyglactin 2/0 sutures. A methylene blue test was conducted to ensure the patency of the bladder closure, followed by layered closure of the abdominal wound.

Following the surgery, the patient exhibited a satisfactory postoperative recovery and remained afebrile. There was a minor episode of hematuria, which resolved spontaneously within two days without the need for bladder irrigation. The renal profile improved to normal levels (urea: 4.6 mmol/l, creatinine: 95.1 umol/l) and acidosis resolved (pH: 7.45, bicarbonate: 22.9 mol/l, base excess: -0.5 mol/l) within one week after the procedure, and the patient was discharged in a stable condition with a suprapubic catheter in place.

One month later, the patient returned to the clinic for a follow-up. During the visit, it was noted that the patient was in good health, consistently adhering to the scheduled suprapubic catheter (SPC) replacements, and displaying a well-healed abdominal scar.

## Discussion

Prostatic urethral stones can be categorized into two main types: endogenous (primary) and exogenous (secondary). Primary stones are typically small and numerous, originating de novo. They form due to the precipitation of substances found in prostatic secretions, which occurs as a result of stagnation caused by obstructions, inflammation, and chronic infection in the prostatic ducts. On the other hand, secondary stones are formed in the upper tract and migrate downwards, which tend to be larger in size [1].

The prevalence of prostatic calculi varies significantly. A study conducted on a Greek population by Geramoutsos et al. reported a relatively low prevalence rate of around 7.4% [2]. In contrast, another study by Kim et al. found a much higher prevalence of 69.97% among patients with benign prostatic hyperplasia (BPH) in South Korea [3]. The majority of cases involve microlithiasis and are observed in males aged 50 years or older. Giant prostatic calculi are exceptionally uncommon and typically occur in conjunction with bladder outlet obstruction [4], as evidenced in our particular case.

Occasionally, patients with prostatic calculi may experience symptoms such as hematuria, recurrent urinary tract infections, dribbling, and incontinence. Prostatic calculi have the potential to not only extend the duration of troublesome symptoms but also reduce the effectiveness of antibacterial treatment in individuals with chronic prostatitis. Patients with prostatic calculi often experience more severe lower urinary tract symptoms (LUTS). Several studies have suggested that the presence of moderate to significant prostatic calculi can be a contributing factor to the development of moderate to severe LUTS [5]. 

Prostatic calculi are frequently linked to urethral or bladder outlet obstruction, with literature indicating that approximately 60% of patients have long-standing urethral stricture disease. Over time, these calculi can grow in size, developing into giant calculi due to additional calcium phosphate deposition in the prostatic urethra. If left untreated, they can lead to the formation of bladder stones due to urine stasis [1]. In certain instances, bladder stones can reach a similar size as prostatic calculi or even larger, as observed in our specific case.

In clinical practice, giant prostatic calculi can be detected during a DRE as a firm and nodular prostate, often mimicking the characteristics of prostatic cancer. The diagnosis of prostatic calculi can be easily made using standard radiographs and ultrasound, with transrectal ultrasound (TRUS) being the preferred modality due to its higher sensitivity [4]. Plain CT KUB is also valuable for evaluating the calculi, mapping them for surgical planning, and ruling out the presence of ureteral stones that could contribute to obstructive uropathy. Cystoscopy, being the gold standard of diagnosing bladder calculus [6], may also help to visualize the prostatic calculi, however it may also just appear as a normally enlarged prostate due to the overlying prostatic urethral mucosa. 

Giant prostatic calculi can be treated using various methods, including transurethral approaches, open retro-pubic surgery, radical prostatectomy, and endoscopic procedures, depending on their location and size. A previously reported case by Kalathia et al. demonstrated successful removal of both giant prostatic calculi and bladder calculus through an endoscopic approach, resulting in a positive outcome [1]. Open surgery offers the advantage of shorter operative time, particularly when dealing with multiple giant stones. In all documented cases where large bladder stones measuring 6.5 cm or more were associated with renal impairment, open cystolithotomy was the chosen treatment method [7]. In our case, we also decided to employ the same approach for stone removal due to the elevated density of both the prostatic and bladder stones, in addition to the aforementioned reasons.

Patients with a large obstructive bladder stone and renal impairment should be closely monitored for postobstructive diuresis during the early stages of the postoperative period. It is advisable to involve renal physicians in such cases for their input and expertise. The recovery of renal function following stone removal is contingent upon the degree of renal damage [7]. Previous literature by various authors reporting cases of giant bladder stones accompanied by renal failure consistently indicated an improvement in renal function after cystolithotomy. While some cases achieved complete recovery of renal function, others experienced partial recovery. In our particular case, renal function fully returned to its normal baseline.

Once the patient has recuperated from the surgery, it becomes crucial to explore potential factors that may contribute to the formation of bladder stones. It is important to address the underlying pathological process in order to prevent the recurrence of stones. In our case, the probable predisposing factor for stone formation was the patient's underlying neurogenic bladder resulting from spina bifida, a well-known associated factor [8]. Therefore, efforts should be made to address and rectify this underlying condition to minimize the risk of stone recurrence.

## Conclusions

The occurrence of giant prostatic calculi alongside a substantial bladder calculus is infrequent and can sometimes lead to obstructive voiding and acute renal impairment. While their diagnosis is straightforward, urgent intervention is necessary to prevent permanent kidney damage. Various management techniques have been described with the common objective of completely removing the calculi and restoring renal function. Careful identification of the underlying cause is crucial in order to prevent recurrence.
